# Molecular Classification of Knee Osteoarthritis

**DOI:** 10.3389/fcell.2021.725568

**Published:** 2021-08-27

**Authors:** Zhongyang Lv, Yannick Xiaofan Yang, Jiawei Li, Yuxiang Fei, Hu Guo, Ziying Sun, Jun Lu, Xingquan Xu, Qing Jiang, Shiro Ikegawa, Dongquan Shi

**Affiliations:** ^1^State Key Laboratory of Pharmaceutical Biotechnology, Division of Sports Medicine and Adult Reconstructive Surgery, Department of Orthopedic Surgery, Nanjing Drum Tower Hospital, The Affiliated Hospital of Nanjing University Medical School, Nanjing, China; ^2^Laboratory for Bone and Joint Diseases, RIKEN Center for Integrative Medical Science (IMS, RIKEN), Tokyo, Japan

**Keywords:** knee osteoarthritis, molecular classification, pathophysiology, body fluids, subtypes

## Abstract

Knee osteoarthritis (KOA) is the most common form of joint degeneration with increasing prevalence and incidence in recent decades. KOA is a molecular disorder characterized by the interplay of numerous molecules, a considerable number of which can be detected in body fluids, including synovial fluid, urine, and blood. However, the current diagnosis and treatment of KOA mainly rely on clinical and imaging manifestations, neglecting its molecular pathophysiology. The mismatch between participants’ molecular characteristics and drug therapeutic mechanisms might explain the failure of some disease-modifying drugs in clinical trials. Hence, according to the temporal alteration of representative molecules, we propose a novel molecular classification of KOA divided into pre-KOA, early KOA, progressive KOA, and end-stage KOA. Then, progressive KOA is furtherly divided into four subtypes as cartilage degradation-driven, bone remodeling-driven, inflammation-driven, and pain-driven subtype, based on the major pathophysiology in patient clusters. Multiple clinical findings of representatively investigated molecules in recent years will be reviewed and categorized. This molecular classification allows for the prediction of high-risk KOA individuals, the diagnosis of early KOA patients, the assessment of therapeutic efficacy, and in particular, the selection of homogenous patients who may benefit most from the appropriate therapeutic agents.

## Introduction

**FIGURE 1:**
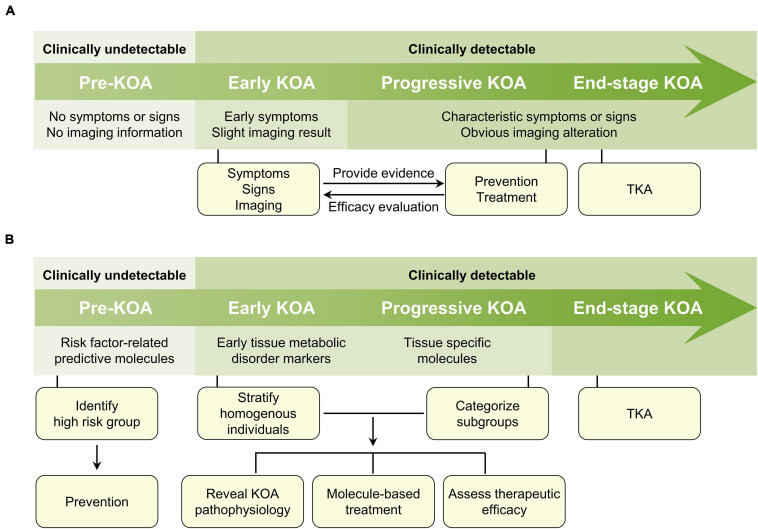
Clinical information-based and molecule-based diagnosis and treatment algorithm of KOA. **(A)** In the clinical information-based KOA diagnosis and treatment algorithm, there is no symptoms, signs, and imaging information for pre-KOA. In early KOA, early symptoms and mild imaging results can be acquired. In progressive and late-stage KOA, the diagnosis is comparatively easy due to obvious clinical manifestations. Treatment decision making principally depends on clinical appearances and in turn, the evaluation of therapeutic efficacy is largely based on clinical outcomes. **(B)** In the molecule-based KOA diagnosis and treatment algorithm, the detection of KOA risk factors-related molecules in body fluids provides opportunity for the prediction of high-risk KOA individuals, and subsequently the preventive strategies. Although clinical information in early and progressive stage are acquirable, disease corresponding molecules permit the stratification of homogenous individuals and the classification of KOA into several subtypes. The molecular classification of KOA may help to reveal the pathogenesis of KOA, explore the molecule-based treatment, and further assess the therapeutic efficacy of treatment. For end-stage KOA, TKA might still be the best option. KOA, knee osteoarthritis; TKA, total knee arthroplasty.

Osteoarthritis (OA) is a common and disabling condition globally, within which knee OA (KOA) accounts for a large proportion and manifests several symptoms that weaken the quality of life, such as pain, stiffness, dysfunction, and even deformity (Martel-Pelletier et al., 2016). Combined with imaging methods, diagnosis for KOA is made on the basis of symptom assessment and a brief physical examination (Hunter and Bierma-Zeinstra, 2019). Plain radiograph is widely used to assess the Kellgren-Lawrence (KL) composite score, and magnetic resonance imaging (MRI) is usually performed to assess the cartilage, synovium and subchondral bone lesions. However, these symptomatic, physical and imaging methods are insensitive to reflect early pathophysiology (Menashe et al., 2012). Besides, current treatment of KOA highly relies on the identification of clinical information and, consequently, the evaluation of therapeutic efficacy is largely based on clinical, and frequently rater-dependent, outcomes (Figure 1A).

Among the drugs applied clinically, some of them show acceptable outcomes in pain relief and joint function improvement, whereas, a fair portion of them with unclear results (Sharma, 2021). Clinical guidelines regarding the use of some pharmaceutics are usually inconsistent (Nguyen et al., 2016). This divergence may be due to the differences between clinical trials, in which the biologically heterogeneous subjects are included. In addition to the completed clinical trials, the inclusion criteria for ongoing clinical trials starting from 2016 to 2021 to explore the effects of a certain drug on KOA did not consider the molecular characteristics of participants (Table 1). Hence, a critical research gap in the matching of drug mechanisms with patients’ molecular features still exists and needs to be filled.

**TABLE 1:** Ongoing clinical trials investigating the efficacy of drugs against KOA.

Targeted pathological process	Drug name	Pharmacological mechanism	Phase of clinical trial	Clinical trial number	Country	Start year
Pain	Fasinumab	Block NGF	I	NCT03491904	United States	2019
	MT-5547		III	NCT03245008	Japan	2017
	LEVI-04	Modulate NGF pathway	I	NCT03227796	United Kingdom	2017
	Cannabidiol	Antagonize CB1, CB2 and GPR55 receptor	IV	NCT04607603	Australia	2020
			III	NCT03825965	Canada	2020
	Duloxetine	Inhibit serotonin and norepinephrine reuptake	IV	NCT04117893	China	2020
	CNTX-4975-05	Activate TRPV1	III	NCT03660943	United States	2018
	Oxytocin	Activate oxytocin, opioid, and cannabinoid receptor	II	NCT04429880	United States	2021
	Bupivacaine	Block the generation and conduction of nerve impulses	III	NCT03838874	United States	2019
Inflammation	Methotrexate	Inhibit NF-κB signaling	N/A	NCT03815448	China	2019
	APPA	Inhibit NF-κB signaling	II	NCT04657926	Denmark	2020
	sc-rAAV2.5IL-1Ra	Gene transfer of IL-1 receptor antagonist	I	NCT02790723	United States	2019
	FX201	Gene transfer of IL-1 receptor antagonist	I	NCT04119687	United States	2019
	PPV-06	Anti-IL-6	I	NCT04447898	France	2020
	Triamcinolone	Corticosteroid	I	NCT04261049	United States	2020
	Zilretta	Corticosteroid	IV	NCT04641351	United States	2020
	TLC599	Corticosteroid	II	NCT03754049	United States	2019
	Dexamethasone	Corticosteroid	IV	NCT03758170	Denmark	2019
	Hydrocortisone	Corticosteroid	IV	NCT04082533	United States	2019
	Triamcort	Corticosteroid	II	NCT02776514	Switzerland	2016
	TLC599	Corticosteroid	III	NCT04123561	United States	2019
	ATB-346	NSAID	II	NCT03978208	Canada	2019
	Lorecivivint	Inhibit Wnt signaling pathway	III	NCT04520607	United States	2020
	SM04690	Inhibit Wnt signaling pathway	II	NCT03727022	United States	2018
	Resveratrol	Antagonize aryl hydrocarbon receptor	III	NCT02905799	France	2017
	Diclofenac sodium	Inhibit COX1 and COX2	III	NCT04683627	United States	2020
	YYC301	Inhibit COX2	II	NCT03850587	Korea	2019
	Celebrex	Inhibit COX2	N/A	NCT04718649	Korea	2021
	Esflurbiprofen	Inhibit COX	III	NCT03434197	Indonesia	2018
	Teriparatide	Synthesize parathyroid hormone for chondroregeneration	II	NCT03072147	United States	2017
	LNA043 (Modified human ANGPTL3)	Induce chondrogenesis and cartilage repair	II	NCT03275064	United States	2017
Cartilage degradation	Fisetin	Block PI3K/AKT/mTOR pathway	II	NCT04210986	United States	2020
	TG-C	Promote TGFβ1 expression	III	NCT03203330	United States	2018
	Native CT-II^®^	Supply Collagen II	N/A	NCT04470336	India	2020
	ENKO1	Promote cartilage ECM synthesis	N/A	NCT03762408	Spain	2017
	Teriparatide	Synthesize parathyroid hormone for chondroregeneration	II	NCT03072147	United States	2017
	LNA043 (Modified human ANGPTL3)	Induce chondrogenesis and cartilage repair	II	NCT03275064	United States	2017
	Ozone	Stimulate antioxidant endogenous system	III	NCT04426721	Italy	2020
	PB125	Activate Nrf2	N/A	NCT04638387	United States	2020
Bone remodeling	99Tc-MDP	Suppress osteoclast differentiation	N/A	NCT02993029	China	2017

*CB1, cannabinoid 1; CB2, cannabinoid 2; COX, cyclooxygenase; ECM, extra-cellular matrix; GPR55, G-protein-coupled receptor 55; IL-1, interleukin 1; IL-6, interleukin 6; KOA, knee osteoarthritis; N/A, not available; NF-κB, nuclear factor kappa-B; NGF, nerve growth factor; Nrf2, nuclear factor-erythroid 2-related factor 2; NSAID, non-steroidal anti-inflammatory drugs; TGFβ1, transforming growth factor-β1; TNFα, tumor necrosis factor α; TRPV1, transient receptor potential vanilloid 1.*

Indeed, patients with KOA exhibit early molecular and structural changes before the disease shows any clinical manifestations (Goldring and Goldring, 2010). Recently, the Osteoarthritis Research Society International (OARSI) endorsed a new definition of OA, emphasizing the molecular derangement as the primary disorder followed by anatomic, and/or physiologic disarrays (Kraus et al., 2015), which highlights a molecular characterization of the pathological mechanisms responsible for KOA. One of the most imperative contributors of the diagnostic work-up of KOA toward a molecular based identification is the research on molecules acting as biomarkers. Indeed, a large amount of KOA-related molecules are detectable in body fluids (Akul et al., 2019), reflecting KOA pathogenesis. Biochemical analysis of body fluids in hospitals and clinics is frequently employed for effective disease diagnosis as they contain numerous valuable disease information (Sung et al., 2021), which provides the prospect of diagnosing and evaluating KOA at the molecular level.

Herein, according to the temporal alteration of representative molecules in body fluids, we propose a novel molecular classification of KOA (i.e., pre-KOA, early KOA, progressive KOA, and end-stage KOA), with a focus on its role in KOA prediction, diagnosis, and treatment efficacy evaluation. This classification criteria may allow for a molecule-based diagnosis and treatment algorithm (Figure 1B), for instance, facilitating the enrollment of biologically homogeneous patients in clinical trials and potentiate the therapeutic efficacy of disease-modifying drugs in a specific patient cluster.

## KOA: A Molecular Disorder

Traditionally, KOA has been considered as a disorder of articular cartilage. However, the current view is that KOA is a whole-joint disease, or even a systemic disorder since it could be affected by various local and systemic risk factors. The major pathogenesis in KOA contains the molecular crosstalk between articular cartilage, synovium, subchondral bone, meniscus, tendon, muscle and infrapatellar fat pad (IFP) (Loeser et al., 2012; Fan et al., 2021). For instance, the accumulation of M1 macrophage in synovium is responsible for the secretion of proinflammatory cytokines, which facilitates the formation of inflammation microenvironment and aggravates cartilage degradation and synovitis (Robinson et al., 2016; Zhang et al., 2020). A variety of cytokines and adipokines secreted by IFP also participate the local inflammation and contribute to the development of KOA (Zeng et al., 2020). In turn, the debris released from degenerated cartilage can also boost the inflammatory response within the joint (Lambert et al., 2019). Such cross-linked molecular dysregulation is the basis of the visibly pathological events, making a strong case to outline its profile in KOA.

From a practical point of view, less invasive or non-invasive methods to obtain the molecular spectrum will have greater clinical significance. Interestingly, along with intense molecular crosstalk, the opportunity to assess molecular changes is presented by the molecular carrying medium, in which synovial fluid (SF), blood, and urine are included (Sung et al., 2021). SF is a gold standard fluid to identify molecules for KOA due to its intimate relationship with various joint tissues and important role in transmitting and receiving molecular signal in the joint cavity (Ingale et al., 2021). Blood and urine are easily withdrawn and reserve a cluster of molecules reflecting KOA pathogenesis (Attur et al., 2011; Bihlet et al., 2019). Given the widespread use of humoral tests, it would be anticipated to characterize the molecular profile of KOA patients by analyzing SF, blood, and urine.

## Pre-KOA

Although the identification of early KOA has gained widespread popularity in the past decade (Madry et al., 2016), there is growing awareness of the necessity to identify the pre-stage of KOA, where the molecular and cellular processes have kicked into action due to the presence of risk factors but without structural changes. To describe the long transition from healthy to clinically assessable early KOA, Ryd et al. defined pre-KOA as “A knee exhibiting one or many risk factors without pain, normal standing radiographs, no structural changes on arthroscopy or standard MRI, that is, before early KOA can be diagnosed” (Ryd et al., 2015). At this symptom- and imaging-free stage, the corresponding molecules of risk factors warrant special attention in the predictive work-up and in the preventive decision making. In turn, risk factors-related molecules may become a proxy for evaluating the efficacy of preventive interventions.

Obesity is a well-established risk factor for KOA. Lifetime risk of KOA rises with increasing body mass index, and longitudinal study shows that two-thirds of obese adults will develop symptomatic OA (Wluka et al., 2013). Despite the excessive joint loading by weight, increasing data shows that adipokines secreted by white adipose tissue indicate the start of KOA before the emergence of clinical manifestations (Abella et al., 2017). As the precursor of adipokines, leptin has been shown to be related to KOA initiation, and a serum elevation of 5 μg/L is associated with a 30% increased risk of structural KOA in obese participants (Karvonen-Gutierrez et al., 2012; Kroon et al., 2019). Similarly, serum resistin starts to increase even 5 years before the onset of radiographic KOA (Van Spil et al., 2012). There is consistent data showing that both leptin and resistin are responsible for activating innate immune responses and stimulating the expression of inflammatory cytokines, which ultimately result in cartilage and bone metabolic disorder (Conde et al., 2010; Acquarone et al., 2019; Xie and Chen, 2019). Accordingly, based on increased adipokine level, in designated cohorts of asymptomatic individuals with high risk for KOA, weight control to reduce adipose tissue mass should be suggested. Leptin and resistin serum level monitoring may help to assess the volatility risk of KOA. Actually, the reduction of 5% body weight within a 20-week period in overweight KOA patients helps them to experience symptomatic relief (Christensen et al., 2007), but the data on decreasing the initial risk of KOA is lacking.

Previous injury is a predominant joint-level risk factor for KOA initiation. Among the injuries, direct articular cartilage damage, anterior cruciate ligament (ACL) injury, and meniscal tear have been reported to be closely associated with KOA development (Vina and Kwoh, 2018). Both ACL injury and meniscal tear can disrupt the stability of knee, alongside with the disturbance of biomechanics and uneven distribution of mechanical load in cartilage (Englund et al., 2012; Wang et al., 2020). Simultaneously, some biomarkers are produced in response to mechanical stimuli (Chu et al., 2018). Among the candidate molecules for mirroring knee injury, cartilage oligomeric matrix protein (COMP) and C-telopeptide of type II collagen (CTX-II) are the most promising. COMP is a pentameric glycoprotein, highly expressed in hyaline cartilage, and plays a vital role in maintaining chondrocyte proliferation and ECM network integrity (Posey et al., 2018). CTX-II is a well-established biomarker for collagen II (Col-II) breakdown (Lattermann et al., 2016). In a community-based cohort study, the highest quartile level of serum COMP at baseline was correlated with the increased risk of radiographic KOA over 20 years (Kluzek et al., 2015). After running 200 km, COMP in the runners’ serum increased threefold, indicating that COMP could be a sensitive indicator of cartilage damage (Kim et al., 2007). After acute ACL injury, COMP concentration in SF is twofold increased within 6 weeks after injury and remains elevated in 5-year follow-up (Struglics et al., 2018). Similarly, the level of CTX-II in SF is significantly increased immediately after ACL injury (Lattermann et al., 2016). Therefore, given that post-traumatic KOA begins at the time of injury (Lattermann et al., 2016), the combination of injury history and elevated COMP and/or CTX-II levels might be a sensitive and effective index in predicting injury-related pre-KOA.

Female sex is another major causal risk factor of KOA, with a prevalence 1.2–2.8 times higher than males (Hunter and Bierma-Zeinstra, 2019; Sasaki et al., 2020). Therefore, interest is growing in the relationship between sex hormones and KOA. In a study enrolling 842 women, Sowers et al. (2006) found that the concentrations of estradiol and its metabolite, 2-hydroxyestrone, in the lowest tertile were strongly associated with higher KOA prevalence and incidence. Considering the protective role of estrogen in cartilage and bone homeostasis, estrogen-related drugs may be favorable for postmenopausal patients against KOA onset, but further preclinical and clinical studies are needed to confirm this (Xiao et al., 2016).

Although KOA is not considered as an autoimmune disease, activated molecular and cellular processes may stimulate maladaptive repair responses, which often include pro-inflammatory pathways of innate immunity and subsequent production of autoantibodies (AAbs) (Leslie et al., 2001; Geurts et al., 2018). Recently, Camacho-Encina et al. (2019) found that the serum level of methionine adenosyltransferase two beta autoantibody (MAT2β-AAb) increased as early as 8 years before the incidence of radiographic KOA. They further verified that the addition of MAT2β-AAb improved the efficiency of clinical prognostic model to identify high risk KOA individuals (Camacho-Encina et al., 2019). This could be applicable in KOA prediction. In addition, although preliminary data suggest that dietary supplementation of the product of MAT2β, S-adenosylmethionine (SAMe), can relieve KOA pain (Kim et al., 2009), further studies are needed to determine whether SAMe has a preventive effect on pre-KOA, especially in people with relatively high serum MAT2β-AAb levels.

Taken together, these predictive molecules will help to discriminate individuals with high risk of KOA initiation (pre-KOA) and may suggest the molecular based interventions to prevent KOA occurrence.

## Early KOA

Luyten et al. (2012) proposed the definition and classification of early KOA, in which physical examination and imaging findings were included. In detail, early KOA should fulfill the following three criteria: (i) knee pain; (ii) KL grade < 2; (iii) cartilage lesions by arthroscopy or MRI, or meniscal or subchondral lesions by MRI (Luyten et al., 2012). Early KOA is thought to be a complicated phase, with limited and sporadic signs or symptoms, usually without early radiographs in most cases. Among this condition, molecules in body fluids can objectively provide useful diagnostic and prognostic information by mirroring the disease relevant biological activity (Mobasheri et al., 2017).

The molecular disorder is a consequence of disturbed gene expression landscape, which is fine tuned by small, non-coding RNAs named microRNAs (miRNAs). By inhibiting the function of protein-coding transcripts, miRNAs alter multiple aspects of cell structure and function, including chondrocytes phenotype (Swingler et al., 2019). Dysregulation of the miRNAs system, driving the disturbances of molecular composition, has been shown as an early detectable mechanism underlies KOA.

Among the miRNAs in early KOA, miR-140 and miR-210 are probably the most representative. Increasing evidence shows that miR-140 is capable for maintaining both cartilage and bone formation and homeostasis (Luo et al., 2018; Swingler et al., 2019). Also, it mediates the inhibition of IL1-induced proteinases (Si et al., 2019). However, the expression levels of miR-140 in SF is significantly reduced in early KOA patients compared with healthy individuals (Si et al., 2016). Furthermore, the SF levels of miR-140 were negatively correlated with the KL grades (Si et al., 2016), suggesting that the protective effect of miR-140 continued to be lost. miR-210 has been recognized as a major hypoxia-induced miRNA that contributes to the induction of angiogenesis (Bavelloni et al., 2017). Xie et al. found that miR-210 was significantly upregulated in patients with early-stage and late-stage KOA compared with healthy subjects (Xie et al., 2019), suggesting that the beginning of an increase in miR-210 level was an indicator of early KOA. These findings allow for early molecular diagnosis of KOA by detecting miRNAs. Other differentially expressed miRNAs, such as miR-19, miR-122, miR-146a, miR-186, miR-210, miR-223 and miR-486, are potential biomarkers of early KOA and still warrant further studies (Kong et al., 2017; Xie et al., 2019; Rousseau et al., 2020). Additionally, the development of disease-modifying drugs, especially miRNA therapy, might be attributed to a well-developed miRNA spectrum in early-KOA.

Another highly promising SF biomarker of early KOA is IL-17. By inducing *de novo* gene transcription or stabilizing target mRNA transcripts, IL-17 upregulates inflammatory gene expression, including IL-1β and TNFα, two major players of KOA pathophysiology (Kapoor et al., 2011; Amatya et al., 2017). The level of IL-17 in SF of KOA patients was significantly higher than that of healthy individuals, but there was no statistically significant difference between KOA groups based on KL grades (Liu et al., 2015). In addition, IL-17 level tended to decrease as the severity of KOA increased. This dynamic signature of changes in IL-17 level may herald that the start of IL-17 elevation indicates the onset of KOA. These characteristics represent the potential efficacy of IL-17 in providing early biological fingerprint of KOA, allowing for early diagnosis and identification of the cluster with inflammation-based KOA onset. Accordingly, the therapeutic effect of IL-17 inhibitors, such as the widely used secukinumab (McInnes et al., 2020), may be strengthened in selected clusters of early KOA individuals with high IL-17 levels, which has not been tested in clinical trials yet.

In addition to IL-17, IL-15 is also detected and viewed as a biomarker for early KOA (Scanzello et al., 2009). Produced by several cell types within the knee, including fibroblasts and macrophages, IL-15 has been shown to stimulate matrix metalloproteinase (MMPs) production, specifically MMP1 and MMP9 (Constantinescu et al., 2001; Waldmann et al., 2001). It was observed that the level of IL-15 in SF was significantly increased in early KOA and decreased in late KOA (Scanzello et al., 2009). Moreover, serum IL-15 levels were significantly higher in KOA patients compared with healthy individuals, but there was no significant correlation with KL grads (Sun et al., 2013). These findings suggest a potential role for IL-15 in the diagnosis of early KOA. Further studies focused on evaluating the utility of IL-15 in clinical practice and delineating molecular pathways responsible for IL-15 secretion are essential.

The identification of a comprehensive molecular profile for early KOA, including miRNAs, IL-17, IL-15, and future identified molecules, provides insight into the pathogenesis of KOA initiation. This strategy could promote the pathological mechanism-based diagnosis and facilitate the molecule-based treatment of early KOA.

## Progressive KOA

**FIGURE 2:**
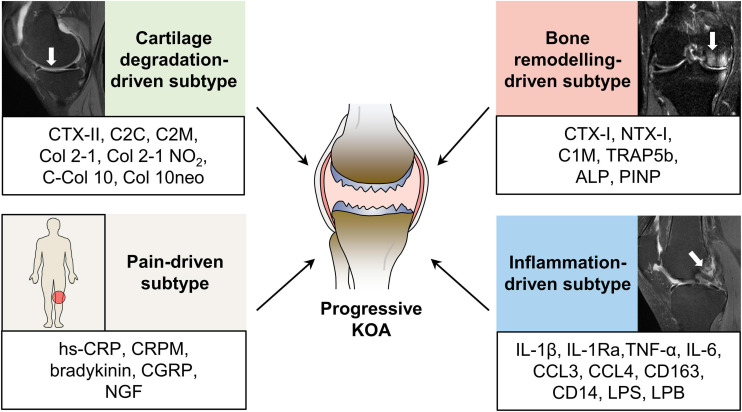
Molecular subtypes of progressive KOA. Increasing evidence suggests that progressive KOA patients fall into several subtypes based on the identified molecular profiles, including cartilage degradation-driven subtype, bone remodeling-driven subtype, inflammation-driven subtype, and pain-driven subtype. Representative molecules are listed in the box bellow the MRI manifestation of each subtype. ALP, alkaline phosphatase; CCL3, CC-chemokine ligand 3; CCL4, CC-chemokine ligand 4; Col 2-1 NO_2_, the nitrated form of Col 2-1; C-Col 10, C-terminus of collagen X; Col 10neo, a neoepitope of collagen 10; CGRP, calcitonin gene-related peptide; CRPM, the fragment of C-reactive protein; CTX-I, C-telopeptide of Col-I; CTX-II, C-telopeptide fragments of Col-II; C2C, the cleavage neoepitope of collagen II; C1M, the product of collagen I degraded by matrix metalloproteinases; C2M, the fragments of collagen II degraded by matrix metalloproteinases; hs-CRP, high sensitive C-reactive protein; IL-1β, interleukin 1β; IL-1Ra, IL-1 receptor antagonist; IL-6, interleukin 6; KOA, knee osteoarthritis; LPS, lipopolysaccharide; LPB, LPS binding protein; MRI, magnetic resonance imaging; NGF, nerve growth factor; NTX-I, N-telopeptide of Collagen I; PINP, N-terminal collagen type I extension propeptide; TNFα, tumor necrosis factor α; TRAP5b, tartrate resistant acid phosphatase 5b.

Knee osteoarthritis is a heterogeneously progressive disease with different clinical phenotypes that eventually leading to a common final pathway of joint destruction (Castañeda et al., 2014). Persistent pathological factors contribute to the development of KOA from an early preventive stage toward an advanced, probably irreversible stage. Unlike accelerated KOA, which initiate and progress into advanced stage within 4 years (Driban et al., 2019), typical KOA is a slow-progressing disease, a process that usually takes decades. Whereas, the heterogeneous progression trajectories of KOA patients present as an obstacle in the development of disease-modifying drugs and the design of clinical trials (Halilaj et al., 2018). Furthermore, the current diagnosis and treatment strategies for KOA are still “one rule applies to all patients” (Yuan et al., 2020), highlighting the necessity for a more accurate molecular classification to provide evidence for targeted therapies. Hence, considering the characteristics of patients with KOA and the classification criteria for better clinical decision making (Dell’Isola and Steultjens, 2018), we classified progressive KOA into four subtypes based on the identified representative molecular profiles: cartilage degradation-driven, bone remodeling-driven, inflammation-driven, and pain-driven subtype (Figure 2). The pathogenesis-based first treatment options for different subgroups are also suggested (Table 2), but more solid data is indispensable to examine the correspondence between the altered molecules and therapeutic drugs.

**TABLE 2:** Potential beneficial treatment options for progressive KOA subtypes.

Progressive KOA subtypes		Treatment principle	Main altered molecules	Potential beneficial drugs
Cartilage degradation-driven subtype		Cartilage extra-cellular matrix components supplement	CTX-II, C2M, C2C, Coll 2-1, C-Col 10	Hyaluronic acid (Bannuru et al., 2011), Glucosamine (Fransen et al., 2015), Chondroitin (Fransen et al., 2015), Undenatured collagen II (Lugo et al., 2016)
Bone remodeling-driven subtype	Bone resorption	Anti-resorption	CTX-I, NTX-I, C1M, TRAP5b,	Bisphosphonate (Aitken et al., 2018), Osteoprotegerin (Sagar et al., 2014), Calcitonin (Karsdal et al., 2015), MIV-711 (Conaghan et al., 2019)
	Bone formation	N/A	ALP, PINP	N/A
Inflammation-driven subtype		Anti-inflammation	IL-1β, IL-1Ra, TNFα, IL-6, CCL3, CCL4	IL-1 inhibitor (Jotanovic et al., 2012), TNFα inhibitor (Grunke and Schulze-Koops, 2006), COX2 inhibitor (Hochberg et al., 2016), NSAIDs (Bannuru et al., 2019)
Pain-driven subtype		Analgesia, Anti-inflammation	hs-CRP, CRPM, bradykinin, CGRP, NGF	Opioids (Kolasinski et al., 2020), NSAIDs (Bannuru et al., 2019), NGF inhibitor (Berenbaum et al., 2020), CGRP inhibitor (Jin et al., 2018), Capsaicin (Kolasinski et al., 2020)

*ALP, alkaline phosphatase; C1M, Collagen I degraded by matrix metalloproteinases; C2C, the cleavage neoepitope of collagen II; C2M, the fragments of collagen II degraded by matrix metalloproteinases; C-Col 10, C-terminus of collagen X; CCL3, CC-chemokine ligand 3; CCL4, CC-chemokine ligand 4; CGRP, calcitonin gene-related peptide; Coll 2-1 NO_2_, nitrated form of Coll 2-1; COX2, cyclooxygenase 2; CRPM, the fragment of CRP; CTX-I, C-telopeptide of Col-I; CTX-II, C-telopeptide fragments of Col-II; hs-CRP, high sensitive CRP; IL-1β, interleukin 1β; IL-1Ra, IL-1 receptor antagonist; IL-6, interleukin 6; KOA, knee osteoarthritis; N/A, not available; NGF, nerve growth factor; NTX-I, N-telopeptide of Collagen I; NSAIDs, non-steroidal anti-inflammatory drugs; PINP, N-terminal collagen type I extension propeptide; TNFα, tumor necrosis factor α; TRAP5b, tartrate resistant acid phosphatase 5b.*

### Cartilage Degradation-Driven Subtype

As the most typical phenotype of KOA lesions, cartilage loss is a dynamic alteration arising from an imbalance between its anabolism and catabolism, leading to changes in the properties of cartilage materials and increases its susceptibility to disruption by physical forces (Hunter and Bierma-Zeinstra, 2019). Detection of debris released from degraded cartilage provides the possibility of assessing cartilage loss during the progression of KOA.

Among the degradation of cartilage components, the most paradigmatic example derives from the disruption of Col-II because it is a major structural and highly specific molecule within cartilage (Martel-Pelletier et al., 2016). Numerous studies have shown that the urinary CTX-II was highly associated with the severity of KOA, mirroring the ongoing Col-II digestion by MMPs and cathepsins (Charni-Ben Tabassi et al., 2008; Xin et al., 2017; Sofat et al., 2019). Besides, CTX-II is an independent risk indicator of total knee arthroplasty (TKA) (Garnero et al., 2020), suggesting potential cartilage-targeting therapy attenuating KOA progression may delay TKA. Of particular interest, the magnitude of change in urinary CTX-II is responsive to the efficacy of conventional treatment measures, such as the chondroprotective glucosamine (Nagaoka et al., 2019). In this scenario, the assessment of CTX-II in urine might allow for the identification of patients who may benefit most from chondroprotective treatments, and CTX-II monitoring may help to evaluate the treatment efficacy dynamically. Notably, urinary CTX-II levels in women have been found significantly higher than in men (van Spil et al., 2013; Bihlet et al., 2019), highlighting its gender differences that need to be critically considered in clinical settings.

Other promising molecules reflecting the extent of Col-II degradation have been extensively explored as progressive KOA biomarkers. The fragments of Col-II degraded by MMPs (C2M) in serum have shown positive association with knee structural changes assessed by KL grades (Siebuhr et al., 2014). In a study enrolling 600 cases, Kraus et al. investigated that the cleavage neoepitope of Col-II (C2C) in urine was remarkably increased in established radiographic KOA individuals, and its time-integrated concentration on 24-month follow-up was predictive for KOA progression (Kraus et al., 2017a). Although detected in different body fluids, both C2M and C2C are suitable for the identification of Col-II degeneration.

After the denaturation of the triple helix of Col-II, Coll 2-1 is released and detectable in urine, as well as its nitrated form, Coll 2-1 NO_2_, which results from the nitration of aromatic amino acids by peroxynitrite under oxidative condition in KOA chondrocytes (Bolduc et al., 2019). In progressive KOA patients, both Coll 2-1 and Coll 2-1 NO_2_ are correlated with the severity of KOA, and their one-year changes are positively associated with joint space narrowing (Deberg et al., 2005), indicating Col-II degeneration and oxidative stress simultaneously happened in KOA pathophysiology. More importantly, the laboratory environment, sampling condition and circadian rhythm have no impact on the measurement of Coll 2-1 (Hick et al., 2019), ensuring Coll 2-1 as a reproducible and credible biomarker for evaluating Col-II degradation. Its responsiveness to treatment, such as intra-articular injection of hyaluronic acid (Henrotin et al., 2013), also warrants its use as an indicator for monitoring therapeutic efficacy.

Another promising strategy for evaluating cartilage degradation is the measurement of molecules reflecting the abnormally enhanced chondrocyte hypertrophy during cartilage destruction (Rim et al., 2020). Type X collagen (Col-X) is a major marker used to detect hypertrophic chondrocyte. In a cluster of 271 KOA patients stratified by KL grade, the serum levels of the C-terminus of Col-X (C-Col 10) were positively correlated with cartilage degeneration (He et al., 2014). In a recent investigation, the higher levels of a neoepitope of Col-X (Col 10neo), ^479^GIATKG, in urine were associated with greater KL scores (He et al., 2019), indicating the ongoing hypertrophic process of chondrocytes during KOA progression. Further validation of C-Col 10 and Col 10neo in large-scale clinical trials is needed with the purpose of their application in clinical practice.

Thus, in progressive KOA, molecules mirroring cartilage degenerative and chondrocyte hypertrophic process help to select cartilage degradation-driven subtype patients, who may benefit most from chondroprotective interventions.

### Bone Remodeling-Driven Subtype

Bone remodeling is a result of the coupling of osteoblastic bone formation and osteoclastic bone resorption (Hu et al., 2021). For mechanically unstable joints, the subchondral bone may exhibit bone bruise, known as bone marrow edema on MRI, which has been shown to be a potent risk factor for disease structural and symptomatic progression in patients with KOA (Felson et al., 2003). Intriguingly, mounting evidence suggests that bone resorption activation mainly occurs in early-stage KOA, whereas, bone formation activation is the major characteristic of late-stage KOA (Funck-Brentano and Cohen-Solal, 2011). Although subchondral bone abnormality is not presented in all KOA individuals, it is indeed the earliest pathological change in a fraction of patients (Hu et al., 2021). Therefore, the identification of molecular profiles in progressive KOA would help to stratify patients to the bone remodeling-driven subtype and provide evidence for the application of pathogenesis-based pharmaceutical medications.

Type I collagen (Col-I) is the most abundant protein in bone, accounting for 90% of total bone protein (Eastell and Szulc, 2017). Attaching to bone with a sealing zone, osteoclasts secrete acid to dissolve the bone mineral (Martel-Pelletier et al., 2016). After that, osteoclasts release enzymes (e.g., cathepsin K) to digest proteins and release the fragments, such as C-telopeptide of Col-I (CTX-I) and N-telopeptide of Col-I (NTX-I), which are indicative of osteoclast activity (Eastell and Szulc, 2017). Several clinical trials with more than 1,000 participants have shown that both urinary CTX-I and serum NTX-I were positively correlated with the symptomatic and radiographic severity of KOA (Kraus et al., 2017b; Bihlet et al., 2019), indicating the activation of bone absorption. In parallel, C1M, the product of Col-I degraded by MMPs, is also suggestive for KOA progression (Siebuhr et al., 2014). Remarkably, monitoring the levels of CTX-I and NTX-I allows for assessing the therapeutic efficacy of targeting bone absorption treatment, as they are significantly reduced in a dose-dependent manner after the treatment of a selective cathepsin K inhibitor, MIV-711 (Lindström et al., 2018). Tartrate resistant acid phosphatase 5b (TRAP5b, also known as ACP5) is one of the osteoclasts produced enzymes. It is fairly specific to bone and its level is responsible for reflecting the number of osteoclasts (Lv et al., 2015). Serum levels of TRAP5b have been shown associated with the severity of knee symptoms in KOA individuals (Nwosu et al., 2017), providing evidence for the aggregation of osteoclasts during bone remodeling. Based on this, KOA patients with obviously active bone absorption may benefit most from the antiresorptive agents, such as bisphosphonates, cathepsin K inhibitor and calcitonin.

Osteoblasts express the highest concentration of collagen during their proliferative phase and of bone alkaline phosphatase (ALP) during matrix maturation (Stein and Lian, 1993). N-terminal collagen type I extension propeptide (PINP), primarily originates from bone, is derived from the post-translational cleavage of type I procollagen and has been well established as a bone formation biomarker (Eastell and Szulc, 2017). Increasing evidence supports that KOA progression, especially the osteophytosis progression, was preceded and accompanied by the enhanced bone formation as assessed by the values of serum PINP (Kumm et al., 2013). Besides, Park et al. (2020) demonstrated that serum ALP activity was independently and positively correlated with KOA severity by including 3,060 participants. The elevation of serum PINP and ALP might be useful in the assessment of abnormal bone formation, such as subchondral bone sclerosis and osteophyte formation, in progressive KOA. In such cases, antiresorptive treatment may be inappropriate and may aggravate the abnormal bone formation. Unfortunately, therapeutic agents targeting the abnormal activation of bone formation has been scarcely investigated.

Several clinical trials have been focused on evaluating the potential therapeutic efficacy of anti-absorption agents on KOA, regrettably, with scarce data on the beneficial effects. For instance, intravenous zoledronic acid, a classical antiresorptive agent, did not show a significant improvement of pain score and the reduction of cartilage volume loss in KOA patients, even in those with bone marrow lesions (Aitken et al., 2018; Cai et al., 2020). The most likely reason for the failure may be that their inclusion criteria were based only on clinical and radiographic diagnosis, which is heterogeneous in molecular level. Further molecular based stratification may help to enroll the biologically homogeneous KOA patients with active bone remodeling and facilitate the highest potential success of the remodeling targeting agents.

### Inflammation-Driven Subtype

Accumulating evidence supports that the inflammation in KOA is chronic, comparatively low-grade, and primarily mediated by the innate immune system (Robinson et al., 2016). Clinically, many patients with KOA have symptoms of joint inflammation, such as pain, morning stiffness, and warmth (Sellam and Berenbaum, 2010). Hence, therapeutic strategies targeting the low-grade inflammation may be able to halt KOA progression. However, disappointing results have been described in several tested anti-inflammatory therapeutics (McAlindon et al., 2017; Deyle et al., 2020). Given the heterogeneity of KOA, revealing the molecular characteristics of inflammation during disease progression might help to recruit homogenous patients for clinical trials testing anti-inflammatory agents.

Highlighting the role of inflammation, much interest has been expressed in identifying secreted inflammatory cytokines in the pathophysiology of KOA progression. The most widely studied biomarkers are IL-1β and TNF-α, two major players of KOA inflammation. Increasing evidence shows that both the serum levels of IL-1β and TNF-α are highly associated with the symptomatic and radiographic progression of KOA (Attur et al., 2011, 2020; Larsson et al., 2015). Their effects can generally be described as the inhibition of cartilage anabolism, activation of cartilage catabolism, and perpetuation of inflammatory responses by inducing the production of other proinflammatory cytokines, such as IL-6 (Livshits et al., 2009; Kapoor et al., 2011). Despite the extensively available non-steroidal anti-inflammatory drugs (NSAIDs) and steroids showing no solid data of restoring the joint damage, anti-IL-1β and anti-TNF-α therapies in several clinical trials represent promising therapeutic efficacy as determined by the relief of pain (Grunke and Schulze-Koops, 2006; Wang et al., 2017). Whereas, the neglection of matching molecular profile with therapeutic mechanisms might be the reason of a recent failure attempting to target IL-1β (Fleischmann et al., 2019). Intriguingly, the plasmatic levels of IL-1 receptor antagonist (IL-1Ra), a natural inhibitor of IL-1, has been shown to be independently associated with the progression of symptomatic KOA (Attur et al., 2015). IL-1Ra possesses anti-inflammatory properties by competitively binding to IL-1 receptors with no signaling transduction effects. The elevation of IL-1Ra may indicate the burden of tissue exposure to inflammation and the endogenous attempt to antagonize the overproduced IL-1β. Techniques delivering IL-1Ra into joint cavity without altering its biological activity may provide new insight for KOA-modifying strategies (Agarwal et al., 2016).

Synovitis is highly associated with KOA progression. Indeed, growing evidence supports the role of immune cells, particularly macrophages, in KOA pathophysiology (Zhang et al., 2020). Some molecules reflecting the infiltration and accumulation of macrophages within the synovium have been proposed as biomarkers of KOA progression (Zhao et al., 2015). The levels of plasma chemokines, such as CCL3 and CCL4, are associated with KOA severity, indicating the infiltration of macrophages and the progression of synovitis in KOA (Zhao et al., 2015). Consequently, the SF levels of CD163 and CD14, two soluble markers for macrophages, are positively associated with the abundance of activated macrophages in synovium (Daghestani et al., 2015), allowing for the timely assessment of macrophage-mediated synovitis progression. The validation of these macrophage-related molecules is needed in view of their application in the development of macrophage targeting therapy.

In addition to local inflammation of the joint, systemic inflammation may also have a vital role in KOA pathogenesis (Huang and Kraus, 2016). For example, obesity is known as an important risk factor of KOA progression possibly not only by the increased mechanical load on the knee joint, but also by the perturbation of the intestinal microbiota and the harvest of persistent and low-grade inflammatory response (Berenbaum et al., 2013; Cox et al., 2015). Weight loss can alleviate KOA symptoms by the substantial reduction of systemic levels of C-reactive protein (CRP) and IL-6, two well-established biomarkers of KOA progression (Beavers et al., 2015). In this context, the assessment of weight loss may represent the decrease of systemic inflammation. Interestingly, lipopolysaccharide (LPS; also known as endotoxin) and LPS binding protein (LBP) are positively associated with the quantity of activated macrophages in knee joint (Huang et al., 2016). In line with this, their levels are also associated with clinical manifestations including total Western Ontario and McMaster University Osteoarthritis Index (WOMAC) scores and self-reported knee pain (Huang et al., 2016).

Identifying the molecular profiles, including the local and systemic inflammatory cytokines, would help reveal the major inflammatory mechanisms and stratify KOA patients with different molecular characteristics. This strategy could facilitate the diagnosis of inflammation-driven subtype of KOA and the development of molecular based treatment strategies.

### Pain-Driven Subtype

Pain is a major driver of health service use and clinical decision making of KOA (Neogi, 2013). However, the origin and mechanisms of pain remain enigmatic. At present, KOA pain is mainly controlled by NSAIDs and analgesics, with unsustainable pain relief and substantial adverse effects (O’Neil et al., 2012). The presence and severity of pain have been shown due to bone marrow lesions and synovitis, and in turn, a change in pain within a person relates to a change in synovitis or in the number or size of bone marrow lesions (Yusuf et al., 2011; Zhang et al., 2011), which may indicate inflammation or bone remodeling-related molecular mechanisms of pain. Therapeutic strategies might vary between patients depending on their underlying disease mechanisms.

Numerous works focused on the anti-inflammatory strategies that might be capable of alleviating KOA pain. Among the inflammatory molecules related to KOA pain, CRP is extensively and long-term used in reflecting the inflammatory response. Jin et al. included 32 studies in their meta-analysis and found that serum high sensitive CRP (hs-CRP) levels were significantly correlated with knee pain and physical function decline, but not with radiographic changes (Jin et al., 2015), indicating that hs-CRP could reflect KOA symptoms progression. Among KOA patients, elevated CRP levels were associated with heightened pain sensitivity (Lee et al., 2011). Of particular interest, the trends of CRP concentrations in serum and SF were consistent and responsive to KOA treatments (Rondanelli et al., 2019). Accordingly, CRP levels may contribute to the selection of patients with inflammatory pain and could monitor the analgesic effect of anti-inflammatory strategies. After degradation by MMPs, the fragment of CRP (CRPM) can be detected in body fluids. CRPM is related to the central pain sensitization and the risk of symptomatic KOA progression (Arendt-Nielsen et al., 2014). However, the levels of CRP and CRPM may be subject to a variety of pathophysiology disturbances, which reduced their specificity to KOA. Future work should address how to improve the specificity of CRP to KOA pain.

Bradykinin is another promising peptide involved in KOA inflammatory pain by exciting and sensitizing sensory nerve fibers (Wang et al., 2005). As a vasodilator and inflammatory nonapeptide, bradykinin is generated in synovium, and its level in SF is related to KOA progression (Bellucci et al., 2013). In KOA patients, intra-articular injection of a specific bradykinin B2 receptor antagonist showed a long-lasting analgesic effect (Meini and Maggi, 2008). Such molecular based analgesic treatment provides an exemplary therapeutic algorithm for KOA pain management.

Recent studies found that several neuronal factors related to bone remodeling played an important role in the innervation of sensory nerves (Martel-Pelletier et al., 2016). During bone absorption, osteoclasts secret H^+^ to induce an acidic microenvironment and then activate the acid-sensing receptor transient receptor potential vanilloid 1, which is responsible for the transcriptional activation of calcitonin gene-related peptide (CGRP), a well-established pain responder (Yoneda et al., 2015). The levels of CGRP have shown to be positively associated with KL grades and the WOMAC pain scores (Dong et al., 2015), suggesting its role in reflecting KOA progression. However, the humanized monoclonal antibody of CGRP, galcanezumab, failed to reduce KOA pain (Jin et al., 2018), which might be a result of the heterogeneity of CGRP levels in their participants. Preosteoclasts can produce nerve growth factor (NGF), which is a key driver of subchondral nerve innervation (Hu et al., 2021). Binding to its high affinity receptor TrKA, NGF excites TrKA+ sensory neurons, leading to the hypersensitivity and hyperexcitability of nociceptors, which is one of the most fundamental mechanisms of clinical pain (Schmelz et al., 2019; Malfait et al., 2020). In the serum and SF of KOA patients, the levels of NGF and TrKA showed a stage-dependent increase in KOA (Montagnoli et al., 2017), indicating their role in mirroring KOA progression. Several studies utilized NGF-neutralizing monoclonal antibodies showed imperative analgesic effects, but with adverse events of unclear etiology (Jayabalan and Schnitzer, 2017; Miller et al., 2017). Further studies should focus on the mechanisms of the side effects produced by NGF blockade therapy.

Collectively, further exploration of the molecular characteristics of KOA pain may allow for the identification of patients belonging to the pain-driven subtype. For these patients, analgesia will be the first treatment option. According to the major molecular mechanisms, the development of specific drugs for pain management will be an excellent approach.

## End-Stage KOA

End-stage KOA has been viewed as a knee with considerable pain and functional limitations, accompanied by structural damage and/or other complications, such as flexion contractures and joint laxity, that prohibit the normal use of a joint (Driban et al., 2016). Generally, end-stage KOA is characterized as KL grade 4 on radiography (Guermazi et al., 2015). For patients at this stage, TKA surgery should be considered if medical interventions failed to improve persistent debilitating symptom (Martel-Pelletier et al., 2016). Although it is easy to diagnose end-stage KOA by symptoms and imaging methods, the value of some molecules in predicting end-stage KOA and evaluating the prognosis of TKA cannot be underestimated.

As aforementioned, KOA is an inflammatory disease with persistent and low-grade inflammation. Among the indicators from blood test, neutrophil-lymphocyte ratio (NLR) has become a useful, economical and simple tool for reflecting inflammation. In a study enrolling 176 KOA patients, blood NLR was significantly higher in the severe (KL grade 4) group (2.18 ± 1.04) than in the mild to moderate group (1.79 ± 0.8), suggesting NLR as an indicator of end-stage KOA (Taşoğlu et al., 2016). Further analysis revealed that NLR ≥ 2.1 was an independent predictor of severe KOA, with a specificity of 77% and a sensitivity of 50% (Taşoğlu et al., 2016). Although these findings are provocative, further longitudinal studies are needed, given that a single blood sample does not provide a stable assessment of NLR.

To explore the relationship between serum miRNA levels and the occurrence of severe KOA, Beyer et al. identified differentially expressed miRNAs in a population-based cohort including 816 individuals (Beyer et al., 2015). They found that let-7e emerged as the most promising predictor of severe KOA necessitating arthroplasty, and that let-7e levels were negatively correlated with the frequency of surgical knee replacement in end-stage KOA (Beyer et al., 2015). This finding may bring light to a new method for the prognostic evaluation of TKA. Further exploration to validate the role of let-7e in different applications, such as KOA pathological mechanism, and disease activity, is warranted to assess its incremental value.

## Future Directions

Given the emerging evidence demonstrating biomarkers for KOA (Mobasheri et al., 2017; van Spil and Szilagyi, 2020), it is conceivable that molecular diagnostic and therapeutic algorithm could be well developed. Based on the representative molecules, classifying KOA patients into different stages and subtypes should logically have an important effect on clinical decision making by presenting the ongoing pathological processes. We clearly recognize that the KOA subtypes are not necessarily mutually exclusive, and sometimes overlap, for example, patients with subchondral bone lesions usually suffer pain (Moisio et al., 2009). However, this does not diminish the clinical significance of molecular classification. A combination of treatments targeting different mechanisms may be effective. Before this, the effect of specific therapies targeting molecular characteristics firstly warrant further validation in longitudinal studies.

In the past decade, MRI has been rapidly evolved due to technical advances, and its application in clinical research has provided sufficient evidence regarding the feature of disease (Link et al., 2003; Li and Majumdar, 2013; Roemer et al., 2020). According to the predominant structural alterations under MRI, KOA has been previously stratified as five different phenotypes (Roemer et al., 2018). However, more accurate information about the pathological characteristics of each phenotype would help to promote the development of disease-modifying KOA drugs. In the future, the combination of clinical parameters, MRI, and molecular information would form a comprehensive diagnosis and treatment algorithm: clinical and imaging characteristics will be used for the initial screening of KOA subtypes, and molecular characteristics will play a dominant role in predicting high risk individuals and determining the drug selection.

In addition, in order to prevent the influence of other diseases, population with similar baseline conditions should be preferred in clinical trials evaluating the KOA molecules. Future studies should also focus on verifying the efficacy and threshold values of a single molecule, or a panel of molecules, in clinical settings, and exploring new molecules to improve the molecular classification to promote its clinical application.

## Conclusion

**FIGURE 3:**
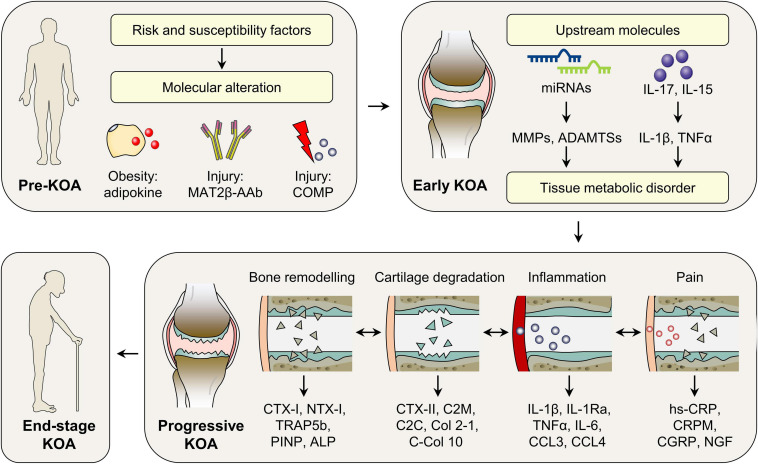
Molecular classification of KOA. According to the temporal alteration of representative molecules during disease initiation and progression, KOA could be classified into pre-KOA, early KOA, progressive KOA, and end-stage KOA. In pre-KOA, risk factors-related molecules, such as adipokines, COMP, and MAT2β-AAb, can be detected in body fluids, which may provide evidence for KOA prediction. In early KOA, molecules can reflect the ongoing pathogenesis, when symptoms and imaging information are ambiguous. Based on the major pathophysiology in patient clusters, progressive KOA is further classified into four subtypes, i.e., cartilage degradation-driven subtype, bone remodeling driven subtype, inflammation-driven subtype, and pain-driven subtype, suggesting different treatment options in future clinical setting. ADAMTSs, a disintegrin and metalloproteinase with thrombospondin motifs; ALP, alkaline phosphatase; CCL3, CC-chemokine ligand 3; CCL4, CC-chemokine ligand 4; C-Col 10, C-terminus of collagen X; CGRP, calcitonin gene-related peptide; COMP, cartilage oligomeric matrix protein; CRPM, the fragment of CRP; CTX-I, C-telopeptide of Col-I; CTX-II, C-telopeptide fragments of Col-II; C2C, the cleavage neoepitope of collagen II; C2M, the fragments of collagen II degraded by matrix metalloproteinases; hs-CRP, high sensitive CRP; IL-1β, interleukin 1β; IL-1Ra, IL-1 receptor antagonist; IL-6, interleukin 6; IL-15, interleukin 15; IL-17, interleukin 17; KOA, knee osteoarthritis; MAT2β-AAb, methionine adenosyltransferase two beta autoantibody; miRNAs, microRNAs; MMPs, matrix metalloproteinases; NGF, nerve growth factor; NTX-I, N-telopeptide of Collagen I; PINP, N-terminal collagen type I extension propeptide; TNFα, tumor necrosis factor α; TRAP5b, tartrate resistant acid phosphatase 5b.

In KOA, different interlinked molecules cause and sustain the pathogenesis, as early as before the clinical and radiographic manifestations are available. As such, based on the representatively investigated molecules, we proposed the novel KOA molecular classification (Figure 3), which offers the possibility to: (i) predict patients at high risk of KOA initiation; (ii) select patients in early and progressive stage, when disease-modifying drugs have the best chance of a successful outcome; (iii) stratify biologically homogenous patients, who may benefit most from therapeutic agents in clinical trials and clinical settings; (iv) provide monitoring biomarkers for the assessment of treatment efficacy; and (v) offer molecular evidence for the development of disease-modifying drugs. Although this molecular KOA classification is merely a simple concept that needs to be further refined, its impact on preclinical and clinical studies is increasing because of the growing need to match molecular mechanisms with treatment strategies.

## Search Strategy and Selection Criteria

We searched titles and abstracts in PubMed, using the search term “osteoarthritis” in combination with, but not limited to, “knee,” “biomarker,” “diagnosis,” “stage,” “progression,” “classification,” “body fluid,” “synovial fluid,” “blood,” “serum,” “urine,” “pathophysiology,” “subtype,” “phenotype,” and “clinical trial.” We mostly selected publications from the past 10 years, although commonly referenced and important older publications were not excluded. English articles presenting data on soluble biomarkers in body fluids (synovial fluid, blood, and urine) of human KOA were included.

## Author Contributions

All authors listed have made a substantial, direct and intellectual contribution to the work, and approved it for publication.

## Conflict of Interest

The authors declare that the research was conducted in the absence of any commercial or financial relationships that could be construed as a potential conflict of interest.

## Publisher’s Note

All claims expressed in this article are solely those of the authors and do not necessarily represent those of their affiliated organizations, or those of the publisher, the editors and the reviewers. Any product that may be evaluated in this article, or claim that may be made by its manufacturer, is not guaranteed or endorsed by the publisher.
